# Exe-Muscle: An Exercised Human Skeletal Muscle Gene Expression Database

**DOI:** 10.3390/ijerph19148806

**Published:** 2022-07-20

**Authors:** Kaiyuan Huang, Jingwen Song, Weishuai Kong, Zhongyuan Deng

**Affiliations:** 1 School of Agricultural Sciences, Zhengzhou University, Zhengzhou 450001, China; huang2420244465@163.com; 2 School of Life Sciences, Zhengzhou University, Zhengzhou 450001, China; songjingwen0704@foxmail.com (J.S.); kongws123@foxmail.com (W.K.)

**Keywords:** Exe-muscle, gene, database, high-throughput sequencing

## Abstract

Human muscle tissue undergoes dynamic changes in gene expression during exercise, and the dynamics of these genes are correlated with muscle adaptation to exercise. A database of gene expression changes in human muscle before and after exercise was established for data mining. A web-based searchable database, Exe-muscle, was developed using microarray sequencing data, which can help users to retrieve gene expression at different times. Search results provide a complete description of target genes or genes with specific expression patterns. We can explore the molecular mechanisms behind exercise science by studying the changes in muscle gene expression over time before and after exercise. Based on the high-throughput microarray data before and after human exercise, a human pre- and post-exercise database was created using web-based database technology, which researchers can use or share their gene expression data. The Exe-muscle database is accessible online.

## 1. Introduction

The advent of high-throughput sequencing technology and the reduction in sequencing costs has led to its widespread use in life science research. This technology has become a conventional technical method for studying gene expression regulation, molecular signal pathways, and determining gene diagnosis target genes [1,2,3]. Some applications of high-throughput sequencing technology include genome sequencing, gene expression analysis, identification of small non-coding RNA, transcription-factor target gene screening, and DNA methylation [4,5,6]. High-throughput sequencing is also applied to sports science research. Gene chip technology is the most used high-throughput sequencing technology to detect gene expression changes [7,8]. Many gene expression chip data related to human movement have been generated in the Gene Expression Omnibus (GEO) database; however, these data have not been fully utilized. Rational and efficient use of these data has also become one of the research objectives.

During movement, the body undergoes adaptive changes according to changes in movement state and time [9,10]. During exercise, people improve the speed, explosive power, and endurance of the corresponding parts, promoting the growth and development of the musculoskeletal system and improving the overall function of the body; these improvements include cardiopulmonary function, metabolism, and nerve response-ability, mediated by repeated contraction and relaxation of muscles and muscle groups. During aerobic exercise, catabolic level increases, and blood flows faster, which is of great significance to the systemic metabolism. Preservation of aerobic fitness and skeletal muscle strength through exercise training can ameliorate metabolic dysfunction and prevent chronic disease. These benefits are mediated in part by extensive metabolic and molecular remodeling of skeletal muscle by exercise. Although both aerobic exercise and resistance training can individually promote substantial health benefits [11], divergent effects are observed depending on the parameter of interest. For example, aerobic training more effectively modifies cardiovascular risk factors, especially in patients with type II diabetes, by affecting total cholesterol, triglycerides, and so on. In contrast, resistance training more effectively maintains basal metabolic rate, muscle mass, and physical function in the elderly [12]. The efficacy of combined aerobic and resistance exercise as part of lifestyle intervention is reflected in the recent exercise guidelines [12]. Improper exercise, including unscientific methods, excessive exercise intensity, inappropriate exercise timing, and environment, produce sports injury and fatigue and even severely damages health and life [13]. These problems require exploration from the molecular perspective, and there remain problems concerning the molecular mechanism underlying motion. Studying the gene expression and signal regulation pathway when adaptive changes occur during sports increases the understanding of the molecular regulation mechanisms. It helps individuals choose appropriate sports activities, direct proper medical treatment, and avoid unreasonable sports forms.

There are few studies on the molecular mechanisms of myocyte adaptation to exercise. In humans, previous studies showed that single exercise leads to changes in the expression of metabolic genes in muscle cells [14,15], suggesting that gene expression regulation occurs during exercise. Based on other model animals, including rats and mice, long-term repeated exercise can cause changes in gene transcription and has lasting effects on protein expression and function [16,17]. These effects are the molecular basis of adaptation to skeletal muscle training. The changes in skeletal muscle gene transcription lead to several stimuli related to skeletal muscle contraction, various signal kinases in response to these stimuli, and downstream pathways and targets of these kinases [18]. Some genes increase rapidly during exercise, some increase slowly during exercise, and the expression of some does not change or is inhibited [19]. However, the changes in many genes during exercise remain unclear [18,19]. A study found that the influence of genes on the results of cardiovascular exercise is estimated to be 44%. In comparison, genes’ influence on short-term explosive exercise is estimated to be only 10%, which suggests that it is possible to formulate personalized exercise programs according to individual genetic characteristics in the future [20]. Looking forward to the future, our body’s response to the same exercise is slightly different because everyone’s genetic composition is different. It should be possible to identify someone’s genotype and then customize a specific training plan for them to improve the exercise program’s effectiveness.

Gene expression chips are DNA microarrays. The core technology fixes the nucleotides to be detected on chips. The detected samples are hybridized with complementary nucleotide probes. After hybridization, fluorescent signals are generated. The number of corresponding probe target genes in the samples can be determined by detecting the strength of fluorescent signals or current signals [21]. At present, the commercially available gene chips include DNA microarrays for detecting genomic DNA and genotype identification, cDNA-microarrays for detecting gene expression and comparison, miRNA-microarrays for detecting miRNA-related gene regulation mechanisms, lncRNA gene chips for studying lncRNA, high-throughput nucleic acid sequencing chips for gene sequencing combined with particular PCR reaction and microarray detection technology, SNP chips for detecting gene diversity, and gene methylation chips for detecting the degree of gene methylation modification.

In the past decade, gene chips and RNA-seq (RNA sequencing) have been applied in various fields. In the early stage, gene chips were used in much research. The widespread use of DNA microarrays is attributable to the development of probe high-throughput design, probe synthesis, and fluorescence detection technology, which makes it possible to detect thousands of probe targets simultaneously, allowing the realization of flexible and accurate detection of target molecules [22]. With the development of sequencing technology and the associated cost reduction, RNA-seq gradually replaced gene chip technology, but both types of sequence data can be used for data mining. In human sports science, a cDNA-microarray is used to measure the effects of different sports on gene expression [23], changes in genes with time during sports [24], and changes in gene expression in the elderly, children, women, and patients [9,25,26].

Databases are organized collections of structured information or data (generally stored in a computer system in electronic form) controlled by a database management system. Web-based technologies are essential in internet development, and web databases permit mining via a web-based interface. The internet is no longer limited to providing information; it can interactively query databases [27]. The composition of a web-based database is not merely a simple combination of databases and web technology; it has developed and become a model for many websites. These databases are composed of four parts. The bottom layer is the database storing data, middle-ware, a web server, and a user-oriented browser. Users query the relevant content through a browser; the queried content accesses the database through the web server, and the query results are displayed through the web page to complete the retrieval process [28]. Web databases combine data storage with the flexible and convenient characteristics of web technology. The database system, an essential part of data storage on the web, accomplishes a perfect combination of database and network technology. Unlike traditional databases, web databases expand the sharing scope of data resources to any network terminal, distributing processing to balance the use of network resources, facilitating data transmission, communication, and statistical analysis, and significantly reducing the cost of system storage and use. Web database technology is used in governmental public services, enterprise management, e-commerce, and life science research.

Therefore, this study aimed to construct a web database based on the gene expression exercised by human skeletal muscle (Exe-muscle). The Exe-muscle database is a comprehensive web-based database containing human skeletal muscle transcript data before and after exercise. The tag coverage for each time SAGE library is over 150,000 and contains a total of 452,095 SAGE tags derived from exercise times 0, 3, 48, and 96 h. Our Exe-muscle facilitates our understanding of exercise’s influence on skeletal muscle and muscular adaptation by gene expression.

## 2. Materials and Methods

### 2.1. Data Sources

The data were derived from the skeletal muscle sample data of GSE43856 in the GEO database [29]. The data source experiment analyzed the changes in the transcriptome of circulating neutrophils and skeletal muscle from standardized resting conditions at 3, 48 and 96 h after an experimental exercise trial (1 h of cycling followed by 1 h of running) in eight healthy, endurance-trained, male subjects. In the data source experiment, the ages of the eight male subjects were similar (25.0 ± 4.1 years), and their body condition was healthy. Their body mass(kg) and body height(cm) were, respectively, 78.6 ± 7.4 and 184.6 ± 4.6. During the cycling phase, the mean O2 consumption was 40.9 ± 7.5 mL·kg^−1^·min^−1^ (73.7 ± 11.0% V_O2max_), and the mean heart rate was 87.3 ± 10.3% of maximum. The mean O2 consumption during the running phase was 48.1 ± 5.2 mL·kg^−1^·min^−1^ (87.3 ± 8.1% V_O2max_), while the mean heart rate was 95.0 ± 4.4% of maximum.

Blood and muscle samples were taken under standardized conditions at baseline and 3, 48 and 96 h after exercise. Total mRNA was extracted from skeletal muscle tissue, and the samples were divided into four groups (0 h before exercise and 3, 48, and 96 h after exercise). Each group underwent eight biological repetitions. The RNA samples were measured using Illumina HT12 version 3 microarrays (Illumina HumanHT-12 V3.0 expression beadchip) and processed using the Illumina iScan platform.

### 2.2. Data Processing and Website Construction

The expression of several genes in 32 groups of data was homogenized to eliminate the error caused by different sequencing depths of intergroup data. The average and standard deviation of eight data in each group were calculated. We generated corresponding table files in the database according to the gene expression.

Appsever (version 7.5.18, PRIMETON, Shanghai, China) was used to build the website, and the relevant database user interface was configured. The website-making tool was phpcms (version 9, Shenghui Zhong, Beijing, China). The Exe-muscle web page was constructed using hypertext preprocessor language and ran on the Linux system (CentOS 6.4, Red Hat, Raleigh, NC, USA). All data were stored in a MySQL database (version 5.1.66, Oracle, Santa Clara, CA, USA), and the tag sequence was stored in an Excel table, which can be downloaded directly.

### 2.3. Short Time-Series Expression Miner (STEM) Trend Analysis

STEM software was used to cluster genes with coherent changes. A *p*-value < 0.05 was set as the threshold for a significant expression pattern. Then, the different expression patterns of time-series genes were compared.

### 2.4. Kyoto Encyclopedia of Genes and Genomes (KEGG) Pathway Enrichment Analysis of Time-Series Genes

We performed a KEGG pathway enrichment analysis to understand the higher-level functions of the time-series genes. According to the previous STEM analysis, we chose trends 11 and 8 for subsequent pathway enrichment analysis. The time-series genes were determined in terms of metabolism, genetic information processing, environmental information processing, cellular processes, organismal systems, and human diseases.

## 3. Results

### 3.1. Construction of the Exe-Muscle Database System

The exercise gene expression database constructed in this study ran on the Linux operating system, used the Apache server as the web server, stored the data in a table in the MySQL database, used the hypertext preprocessor language to realize the user’s retrieval in the database, and finally, inputted the retrieval object in the web and outputted the retrieval results to the user [28,30].

**Figure 1:**
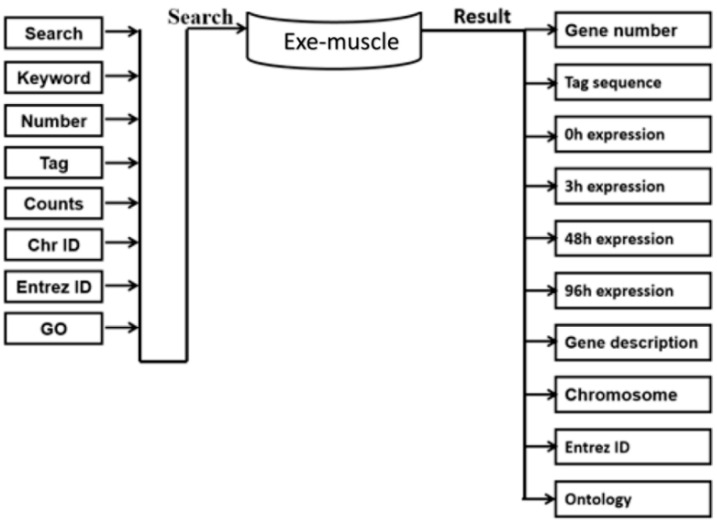
Construction of the Exe-muscle database. The diagram shows the framework of the Exe-muscle database visually. We searched by entering the keyword, number, tag, counts, Chr ID, Entrez ID, or GO (gene ontology) of the gene we wanted to discover. Relevant information was loaded in advance so that we could obtain the search results rapidly: gene number, tag sequence, 0 h expression, 3 h expression, 48 h expression, 96 h expression, gene description, chromosome, Entrez ID, and ontology.

Figure 1 shows the framework of the construction of the Exe-muscle database. First, the gene expression chip of skeletal muscle was downloaded from the GEO database 0 h before exercise and 3, 48, and 96 h after exercise. The data were then processed as the original data in the database. The keywords, UniGene, Tag sequence, Count, Chr, Entrez ID, and Ontology of the retrieval items in the retrieval system were combined for a query. The query displayed gene number, tag sequence, 0 h expression, 3 h expression, 48 h expression, 96 h expression, gene description, chromosome, location information, Entrez ID number, and ontology annotation information.

### 3.2. Using the Exe-Muscle Database to Query the Changes of Related Genes during Exercise

The Exe-muscle database website is a simple and convenient website for users to visit www.Exe-muscle.top (accessed on 1 July 2021) to access. It comprises six parts: a home page, gene retrieval, methods, species, data download, and problem feedback.

**Figure 2:**
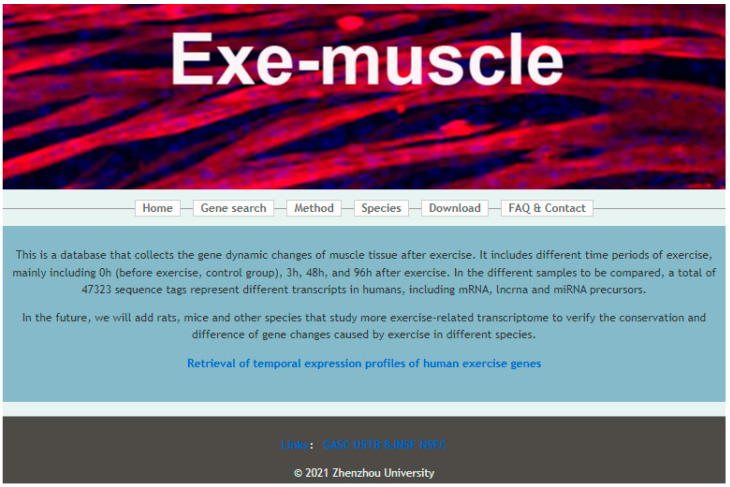
Home page. The row of buttons allows access to the home page, gene search, method, species, download, FAQ, and contact pages. Below is a simple description of Exe-muscle.

**Figure 3:**
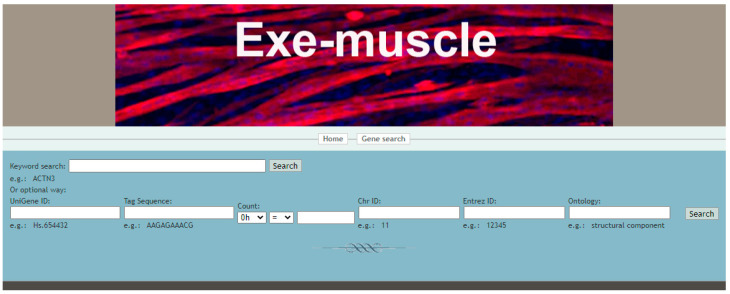
Gene search page of Exe-muscle. This page includes blanks for entering information about the desired gene, such as the Unigene ID, tag sequence, count, Chr ID, Entrez ID, and ontology.

The home page interface includes the home page, the entry buttons of five other functions or web pages, and the introduction to Exe-muscle (Figure 2). Clicking the home button on any web page returns to the home page to enter other functions. The gene search page can query specific genes according to the user’s input to analyze the gene expression at various periods after exercise. Input options include gene name, tag sequence, gene ID, chromosome, gene ontology, and gene expression range (Figure 3). The method page displays a schematic diagram of the construction method of the Exe-muscle database. The species page shows one species currently available for query and two species to be added (mouse and rat). Users can download tag sequence, series data, and chip annotation information on the download page. The FAQ and Contact page provides the website creator’s mailing address and email address for mutual communication and learning.

α-Actinin-3 (ACTN3), also known as the exercise gene, encodes a myofibril protein, fixes different actins and assists in muscle contraction. Previous studies showed that ACTN3 is an essential gene during exercise. The gene’s function relates to the explosive power of skeletal muscle. The proportion of the ACTN3 gene in endurance sports athletes is about 50%, which is not significantly different from that of the general population. By testing, the proportion of normal ACTN3 carried by high-level athletes participating in explosive events (e.g., sprint and long jump) in the Olympic Games was 95%. In comparison, 100% of female athletes in some individual events carried the gene [31,32].

**Figure 4:**
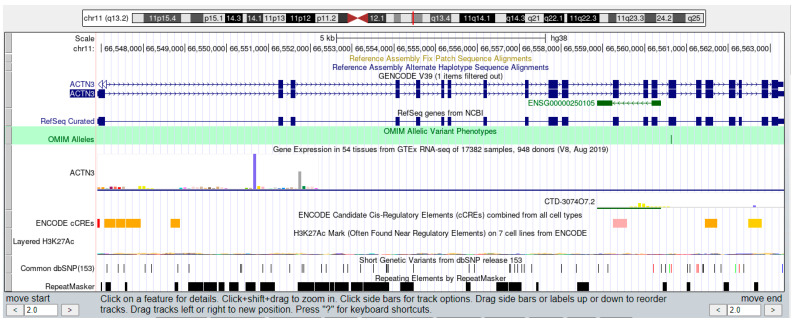
ACTN3 is linked to UCSC. Click the content in Chr to link to the UCSC genome browser, as shown in the figure. Here, it shows a fragment of chromosome 11 ranging from 66,082,191 to 66,092,191.

We use ACTN3 as an example to demonstrate the use method and function of the Exe-muscle database. One can quickly enter the gene search page by understanding Exe-muscle’s overall framework. Directly enter “ACTN3” in the keyword search and click search. ACTN3 appears at the bottom of the page. Its gene number (NM_001104.1) appears with the tag sequence used in the gene chip and the corresponding gene expression (number of reads) at 0 h before exercise and 3, 48, and 96 h after exercise (1811, 2022, 1142, and 1336, respectively). The gene description follows this information as “*Homo sapiens* actin, alpha 3 (ACTN3), mRNA.”, located on chromosome 11, 6,608,719, the Entrez ID number is 89, and the annotation of gene ontology is “A filamentous structure formed of a two-stranded helical polymer of the protein actin and associated proteins. Actin filaments are a major component of the contractile apparatus of skeletal muscle and the microfilaments of the eukaryotic cytoskeleton.”. In addition, the Entrez ID is also set with a hyperlink, which can be clicked and linked to the Genbank database of NCBI. Therefore, the external database NCBI and UCSC (Figure 4) tools can be used for further analysis.

According to the location of the genome, one can link to the UCSC genome browser to further analyze this gene. In addition, 584 miRNAs and about 855 long-chain non-coding RNAs were included in the database.

The expression quantity of the tag sequence can also be retrieved through the retrieval page. This functionality allows searches for genes at the expression level. One can query some genes with low or high expression. Some features can also be combined for retrieval. For example, 3 h after exercise, the number of tag sequences is more significant than 2000, and the gene function is skeletal muscle. On chromosome 11, the target gene ACTN3 in the search result map demonstrates that the gene of a particular feature type can be quickly located.

### 3.3. Using Exe-Muscle to Explore the Change Trend of Genes in Muscle during Exercise

**Figure 5:**
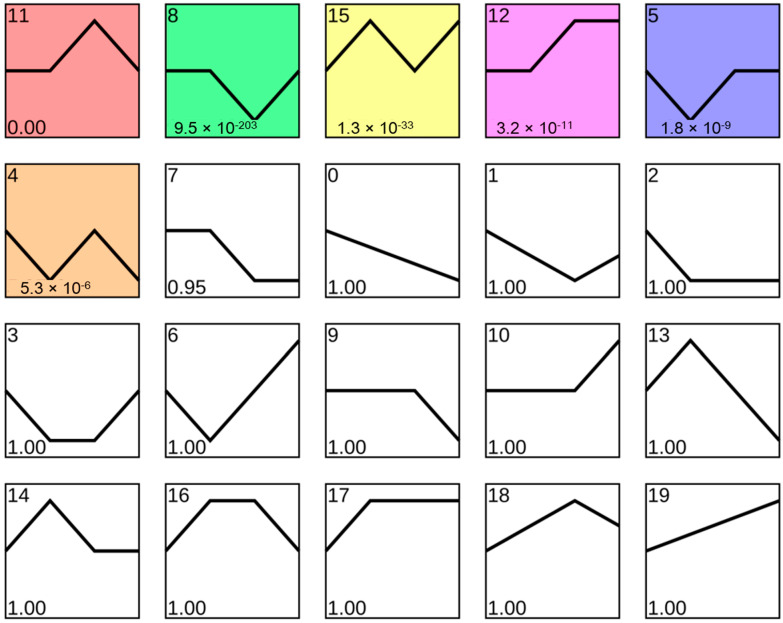
Analysis of the gene trends. We obtained expression patterns of time-series genes at various times. The colored profiles represent significant profiles.

In addition to searching and querying a single gene, the Exe-muscle database can analyze macro genes. When one does not enter the search content and click search, one can obtain the changes of all genes at various times. We used STEM to analyze the change trend of all genes. We obtained 20 genes with different trend change types, of which the first five are gene categories with different changes (Figure 5). We focused on the first two categories, trends 11 and 8. Trend 11 (the change trend) was that the expression level remained unchanged at 3 h, increased at 48 h, and returned to normal levels at 96 h. The category of trend 8 is that it remained unchanged at 3 h. The expression was downregulated at 48 h, and the gene returned to normal levels at 96 h.

We performed KEGG signal pathway analysis on trends 11 and 8 and observed the most genes enriching in human disease pathways on trends 11 and 8. The metabolism and organismal systems pathways were also highly enriched.

**Figure 6:**
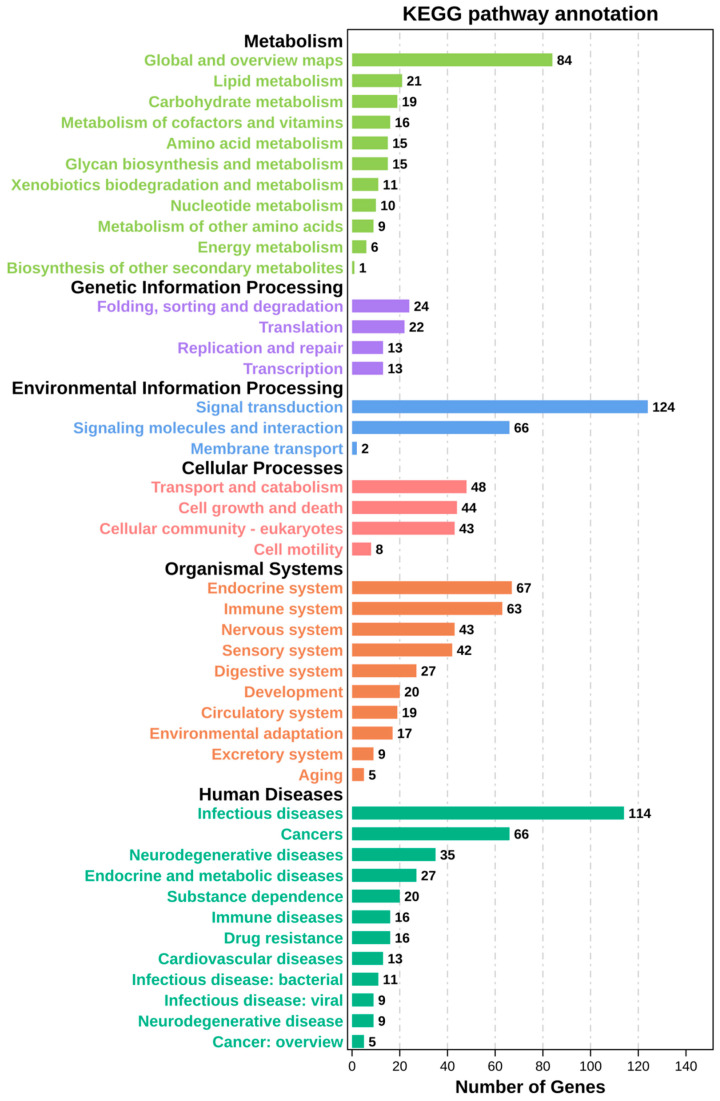
KEGG analysis of trend 11. Bar chart of enrichment of genes in KEGG pathways. The ordinate shows the pathway names in different colors, and the abscissa shows the number of genes in corresponding pathways.

In trend 11, most genes are enriched in signal transduction of environmental information processing, up to 124. The second most is infection diseases, followed by global and overview maps. Others are at similar or lower levels (Figure 6).

**Figure 7:**
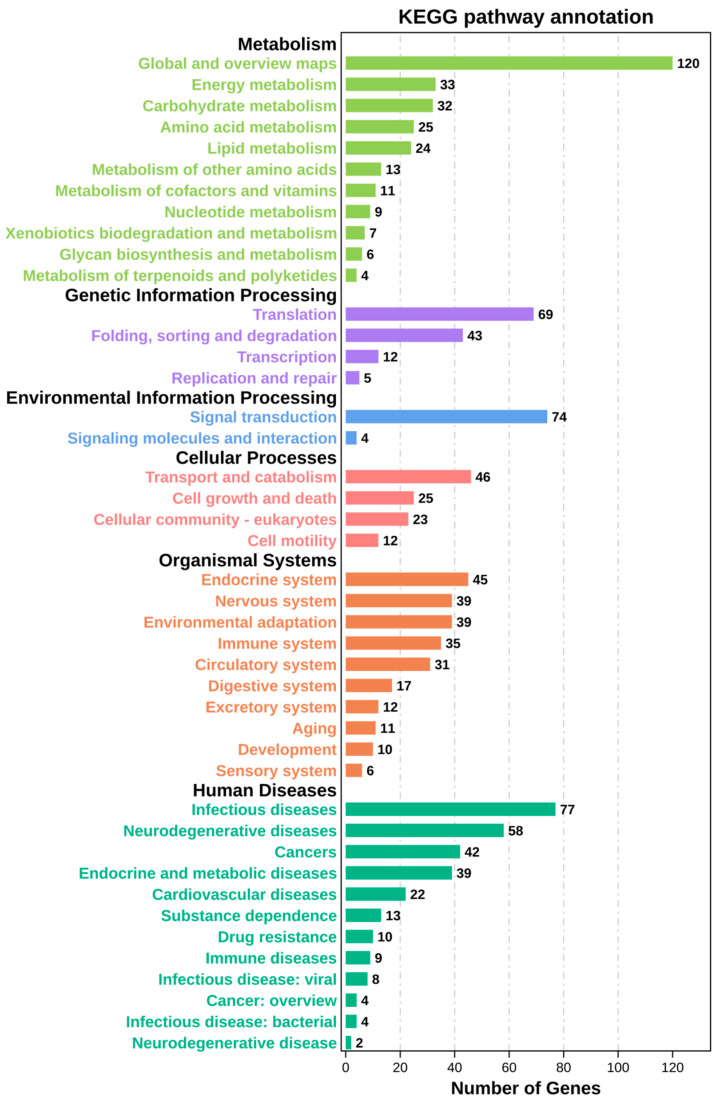
KEGG analysis on trend 8. The form and contents of the chart are in Figure 6. In trend 8, most genes are in the global and overview maps.

In trend 8, most genes cluster in global and overview metabolism maps. Infectious disease signal transduction and translation pathways possess approximately 70 genes. The number of genes enriched in the rest pathways is lower (Figure 7).

## 4. Discussion

We constructed an exercise gene expression database (Exe-muscle) that collects transcripts during and after exercise. It operates on a very flexible platform. In addition to static data analysis, we used SAGE data to extract the information on transcripts expression at different time points after the exercise. The database can be searched for a specific gene to analyze the gene expression pattern, and the total gene expression trend can also be analyzed.

When one types in the gene name, the Unigene ID, tag sequence, description, exact position in the chromosome, Entrez ID, and ontology are returned. More importantly, we can get the reads before and at 3, 48, and 96 h after exercise; this feature is unavailable in most other databases. The user clicks the information under Chr to directly link to the UCSC genome database to view other information about the chromosome, including mRNA and EST. The user then clicks the Entrez ID of the gene to link to the NCBI nucleoside database to view the gene’s description. The Exe-muscle database page is simple and intuitive, and the gene information provided is relatively comprehensive. The disadvantage is that, at this stage, the gene information in the database is limited to humans. Currently, gene information for mice and rats is being developed, and we expect to build an exercise gene expression database containing more species in the future.

The expression level, the number of tag sequences, and other features can be combined and searched to obtain genes that meet the feature requirements. In addition to analyzing a single gene, the Exe-muscle database can conduct macro analysis of several genes. If one clicks ‘Search’ without entering content, changes in all genes at different times are returned. STEM software can analyze the change trends of multiple genes to obtain different gene clusters. In other databases, the length of a protein encoded by gene and GC content are provided [33]; we are considering adding this feature in a future version of our database.

The Exe-muscle exercise gene expression database collected transcripts before and at 3, 48, and 96 h after exercise. More factors would affect gene expression with the change in time after exercise; the study designer tried to make all conditions consistent. The physical condition of the participants could affect the measured index, and as a result, they were required to abstain from any nutritional supplementation and medication throughout the study period. The participants were eight endurance-trained male individuals, nonsmokers who engaged in regular endurance training and had no cardiopulmonary or metabolic disease history. Moreover, they were required to abstain from any nutritional supplementation and medication throughout the study. Furthermore, before each sampling, the subjects were examined by a physician to ensure that they were free of any symptoms of acute illness. Even if we controlled the variables as much as possible, the degree of variation between individuals was still large. Therefore, in the follow-up research, the variation between individuals should also be taken into account in the mechanism of exercise-induced gene expression.

Currently, data are being collected to analyze the dynamic gene expression of different sports types, model animals (rat, mouse), and relevant sequencing data produced [34,35]. This information will be displayed in the next version of the Exe-muscle database. The current version presents the transcript in tables, which is not conducive to intuitive comparison. In subsequent versions, we will increase the graphical display. To analyze other data, we will increase the display of the SAGE data directly on the UCSC genome browser. The long-term goal of Exe-muscle is to serve as a centralized data display and analysis platform to explore and discover muscle cells’ dynamic gene expression changes during exercise and the physiological mechanism behind these changes.

## 5. Conclusions

Existing chip data in the GEO database were screened using a bioinformatics method, and we constructed an exercise gene expression database. The database can be linked to other browsers for further analysis. In addition to analyzing individual genes, the database can analyze macro genes. STEM can be used to analyze the change trend of all exercise genes. We obtained 20 types with different change trends. We focused on the two most significant expression differences, trends 11 and 8. Using KEGG pathway analysis, we found that most genes become enriched in signal transduction of environmental information processing in trend 11 and global and overview maps of metabolism in trend 8. Unlike other signal pathways, human diseases involve several physiological processes, which provide the basis for studying the relationship between disease and exercise. Future study is not limited to increasing valuable information for other essential species; it will also provide bioinformatics tools such as BLAST sequence search in the next version.

## Data Availability

The data presented in this study are available on request from the corresponding author.
